# MEASURING LOST-WORK OPPORTUNITY AT RETIREMENT AGE

**DOI:** 10.1093/geroni/igz038.162

**Published:** 2019-11-08

**Authors:** Maren Wright Voss, Soham Al Snih, Wei Li, Man Hung, lorie Richards

**Affiliations:** 1 Utah State University, Logan, Utah, United States; 2 University of Texas Medical Branch at Galveston, Galveston, Texas, United States; 3 University of Utah, Salt Lake City, Utah, United States; 4 Roseman University of Health Sciences, South Jordan, Utah, United States

## Abstract

There is uncertainty related to whether retirement negatively impacts health--possibly due to the complexity of retirement decisions. The role of lost work opportunity on retirement decisions may help clarify when retirement has a favorable or negative impact on health. Lost work opportunity can be defined as forced retirement or unemployment prompting an earlier than planned retirement. However, 17% of individuals retiring due to the loss of work opportunity (i.e., unemployment, temporary lay-offs, company buy-outs, forced relocations, etc.) do not report either unemployment or involuntary retirement in survey data. We propose a broader conceptualization of late-career unemployment. Using the Health and Retirement Study (HRS), a lost-work opportunity score (LOS) was computed from items indicating unemployment and forced or unplanned retirement. Correlations were computed between this LOS and all continuous variables in the RAND longitudinal compilation of the HRS to determine its convergent and discriminant validity. The LOS demonstrated a Chronbach’s alpha of α=0.82 and had convergent validity with constructs of employment (9 variables), finances (36 variables), and health (14 variables), as predicted by the literature on retirement timing. No other continuous variables in the HRS were identified with a moderate or strong correlation to LOS, demonstrating discriminant validity. Further research should explore whether a combination of variables in the HRS can improve the accuracy of measuring retirement voluntariness. Improved precision in measurement, through an expanded conceptualization of lost-work opportunity, may help explicate the retirement-related factors that impact health, to inform policy and support healthy aging decisions at a societal level.

